# Origins and Properties of Dental, Thymic, and Bone Marrow Mesenchymal Cells and Their Stem Cells

**DOI:** 10.1371/journal.pone.0046436

**Published:** 2012-11-21

**Authors:** Yukiya Komada, Toshiyuki Yamane, Daiji Kadota, Kana Isono, Nobuyuki Takakura, Shin-Ichi Hayashi, Hidetoshi Yamazaki

**Affiliations:** 1 Department of Stem Cell and Developmental Biology, Mie University Graduate School of Medicine, Tsu, Japan; 2 Department of Signal Transduction, Research Institute for Microbial Disease, Osaka University, Suita, Japan; 3 Division of Immunology, School of Life Science, Faculty of Medicine, Tottori University, Yonago, Japan; University of Medicine and Dentistry of New Jersey, United States of America

## Abstract

Mesenchymal cells arise from the neural crest (NC) or mesoderm. However, it is difficult to distinguish NC-derived cells from mesoderm-derived cells. Using double-transgenic mouse systems encoding *P0-Cre*, *Wnt1*-*Cre*, *Mesp1-Cre*, and *Rosa26EYFP*, which enabled us to trace NC-derived or mesoderm-derived cells as YFP-expressing cells, we demonstrated for the first time that both NC-derived (P0- or Wnt1-labeled) and mesoderm-derived (Mesp1-labeled) cells contribute to the development of dental, thymic, and bone marrow (BM) mesenchyme from the fetal stage to the adult stage. Irrespective of the tissues involved, NC-derived and mesoderm-derived cells contributed mainly to perivascular cells and endothelial cells, respectively. Dental and thymic mesenchyme were composed of either NC-derived or mesoderm-derived cells, whereas half of the BM mesenchyme was composed of cells that were not derived from the NC or mesoderm. However, a colony-forming unit-fibroblast (CFU-F) assay indicated that CFU-Fs in the dental pulp, thymus, and BM were composed of NC-derived and mesoderm-derived cells. Secondary CFU-F assays were used to estimate the self-renewal potential, which showed that CFU-Fs in the teeth, thymus, and BM were entirely NC-derived cells, entirely mesoderm-derived cells, and mostly NC-derived cells, respectively. Colony formation was inhibited drastically by the addition of anti-platelet–derived growth factor receptor-β antibody, regardless of the tissue and its origin. Furthermore, dental mesenchyme expressed genes encoding critical hematopoietic factors, such as interleukin-7, stem cell factor, and cysteine-X-cysteine (CXC) chemokine ligand 12, which supports the differentiation of B lymphocytes and osteoclasts. Therefore, the mesenchymal stem cells found in these tissues had different origins, but similar properties in each organ.

## Introduction

All organs consist of layers of epithelial cells derived from one of the germ layers and mesenchymal cells derived from the neural crest (NC) or mesoderm. NC cells emerge from the dorsal region of the neural tube during embryogenesis and differentiate into melanocytes, neurons, glia, and mesenchymal cells, including osteoblasts, chondrocytes, adipocytes, odontoblasts, and perivascular cells [1], [2]. NC cells participate in the organogenesis of the craniofacial area, including the tooth, heart, thymus, and bone marrow (BM) [2]–[7]. In particular, the cephalic NC supplies perivascular cells to the craniofacial area and thymus [8]–[10].

Mesoderm-derived cells have the potential to differentiate into osteoblasts, chondrocytes, and adipocytes, and contribute to the mesenchymal cells in the heart, thymus, and BM [2], [4], [11]. The craniofacial skeleton, including the mandible and maxilla, mainly develops from NC-derived cells; the skeleton outside this region mainly develops from mesoderm-derived cells [2], [12]. Some mesoderm-derived cells contribute to the bones and cartilage of the cranial base and head muscles [13], [14]. Mouse neck and shoulder skeleton is derived from mesenchymal cells that develop from both mesoderm-derived and NC-derived cells [15]. However, it is difficult to distinguish between NC-derived and mesoderm-derived cells.

Mesenchymal stem cells (MSCs) are long-term self-renewing cells, giving rise to one or more specialized cell types [16]. Friedenstein et al. first identified MSCs *in vitro* and termed them fibroblastic colony-forming units (CFU-F) [17]. They defined CFU-Fs as a BM cell population grown in a serum-containing medium that produces colonies of adherent fibroblast-like cells, which can differentiate into osteoblasts, chondrocytes, and adipocytes [17]. Although the origin of MSCs is unclear, they are present in both embryonic and adult tissues in mice and humans [16], [18]–[20]. NC-derived multipotent cells in rodents can differentiate into neurons, glia, and myofibroblasts in the gut and sciatic nerve [21]–[23]; they have potentials similar to MSCs in the skin and BM [6], [24]–[26].

To distinguish NC-derived cells from mesoderm-derived cells, we used double-transgenic mouse systems encoding *P0-Cre*, *Wnt1*-*Cre*, *Mesp1-Cre*, and *Rosa26EYFP*, which enabled us to trace NC- or mesoderm-derived cells as YFP-expressing cells [27]–[32]. *Wnt1* and *P0* are expressed in early migratory NC [5], [30], and *Mesp1*, a transcription factor, is first observed at E6.5 (early gastrulation stages), specifically in nascent mesoderm-derived cells [28]. In this study, we investigated the contributions of NC-derived and mesoderm-derived cells to the teeth, thymus, and BM using three transgenic mouse lines to establish the origin and properties of dental, thymic, and BM MSCs. CFU-F assays indicated that dental, thymic, and BM CFU-Fs comprise NC-derived and mesoderm-derived cells. We clarified the presence of cells in CFU-F progeny with the capacity for repeatable colony formation and retained multipotency.

## Results

### Contributions of NC-derived and mesoderm-derived cells to dental mesenchyme

We used *Wnt1-Cre*, *P0-Cre*, and *Mesp1-Cre* mice crossed with *Rosa26EYFP* mice (i.e., *Wnt1/YFP*, *P0/YFP*, and *Mesp1/YFP* mice, respectively) to investigate the contribution of NC-derived and mesoderm-derived cells to dental mesenchyme. Initially, we isolated hematopoietic cell-deprived YFP^+^ and YFP^−^ cells and examined the gene expression associated with the NC or mesoderm. Approximately two-thirds of YFP^+^ cells from E9.5 *Wnt1/YFP* or *P0/YFP* embryos (i.e., Wnt1/YFP^+^ and P0/YFP^+^ cells) expressed p75NGFR (Fig. S1A). E9.5 Wnt1/YFP^+^ (P0/YFP^+^) and Mesp1/YFP^−^ cells expressed NC-associated genes such as *AP2* and *Sox10* (Fig. S1B). Wnt1/YFP^+^ cells in the dental mesenchyme, which were isolated from E13.5 and two-day-old mice, expressed NC-associated genes such as *p75*, *Sox10*, and *Krox20*, whereas Wnt1/YFP^−^ cells expressed *Brachyury (T)*, a mesodermal gene (Fig. S1C). Therefore, we concluded that *Wnt1-Cre* and *P0-Cre* identified NC-derived cells.

**Figure 1:**
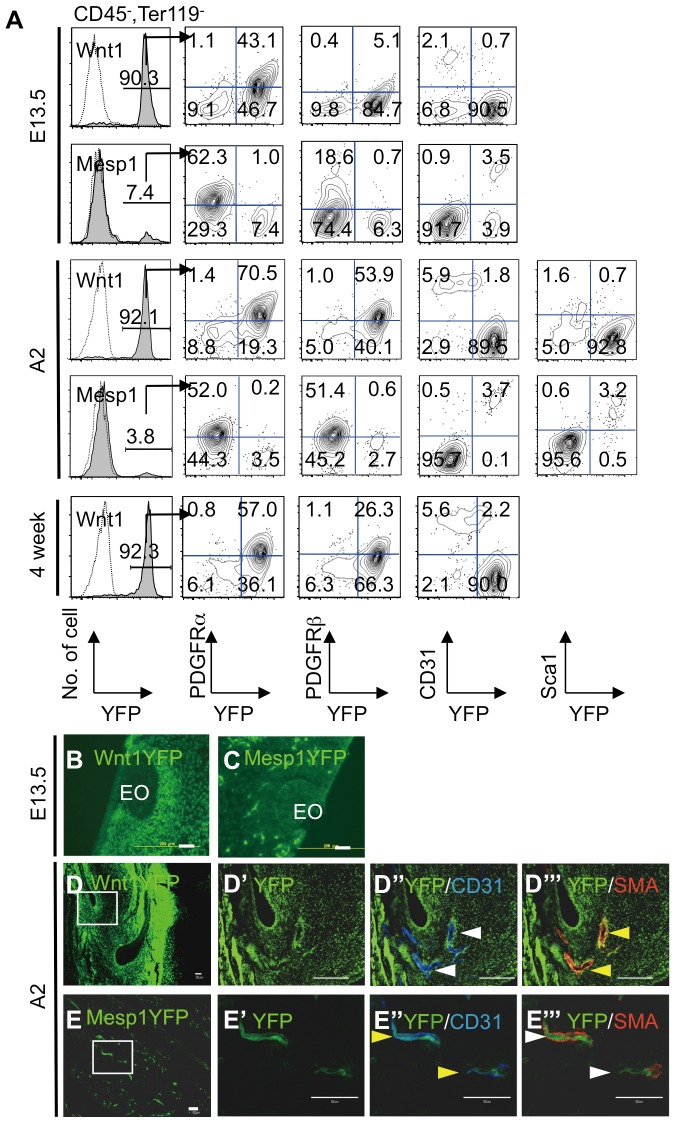
Origins and characteristics of NC-derived and mesoderm-derived cells of the dental mesenchyme. (A) Expression of YFP and cell surface molecules by dental mesenchymal cells prepared from E13.5, 2-day-old, and 4-week-old *Wnt1-Cre/YFP* and *Mesp1-Cre/YFP* mice. The proportions of YFP^+^ cells in the CD45^−^ and Ter119^−^ fractions are indicated. (B, C) Immunohistochemistry of YFP in the mandibular molars of E13.5 *Wnt1-Cre/YFP* (B) and *Mesp1-Cre/YFP* embryos (C). EO, enamel organ. (D, E) Immunohistochemistry of YFP, CD31, and α-SMA in the mandibular incisors of 2-day-old *Wnt1-Cre/YFP* (D) and *Mesp1-Cre/YFP* mice (E). High-magnification views (D′–D′″and E′–E′″) of the boxed areas in (D) and (E), respectively. Yellow and white arrowheads indicate positive cells for each antibody to indicate the presence or absence of YFP^+^ cells, respectively. Scale bars = 50 µm. All experiments were repeated in duplicate and one representative experiment is presented.

To assess the proportion of Wnt1/YFP^+^ cells in the dental mesenchymal cells, we prepared samples from mice that were devoid of blood cells. We found that approximately 90% of dental mesenchymal cells from E13.5 or two-day-old mice were Wnt1/YFP^+^, whereas only approximately 7% were Mesp1/YFP^+^ (Fig. 1A). This difference of approximately 10-fold was observed despite the presence of both NC-derived and mesoderm-derived cells in dental mesenchyme. Large numbers of E13.5 or two-day-old Wnt1/YFP^+^ cells were observed in histological sections of the dental mesenchymal layer around the enamel organ and dental pulp, and Wnt1/YFP^+^ cells were distributed throughout the mesenchyme, whereas only small numbers of Mesp1/YFP^+^ cells were found in these locations (Fig. 1B, C).

### Characteristics of dental mesenchymal cells and the origins of their CFU-Fs

We fractionated dental mesenchymal cells using three markers to compare their origins: CD31 (an endothelial marker), platelet-derived growth factor receptor-α (PDGFRα) (a mesenchymal cell marker), and PDGFRβ (a mesenchymal cell or perivascular cell marker). Among the E13.5 dental mesenchymal cells, Mesp1/YFP^+^ expressed CD31 but Wnt1/YFP^+^ cells rarely expressed it. In contrast, Wnt1/YFP^+^ cells expressed PDGFRα and PDGFRβ but Mesp1/YFP^+^ rarely expressed these markers(Fig. 1A). *Wnt1-Cre* and *Mesp1-Cre* were indicators of reciprocally separable cell populations. PDGFRα- and PDGFRβ-expressing cells were found only in the Mesp1/YFP^−^ cell fraction. Dental pulp cells from two-day-old and four-week-old mice produced similar results (Fig. 1A). We also examined the expression of the endothelial cell markers CD34, FLK1, and Sca1 (an MSC marker). Sca1 was expressed in Mesp1/YFP^+^ cells from two-day-old mice (Fig. 1A). All four-week-old Mesp1/YFP^+^ cells expressed CD31, whereas 42% and 53% expressed CD34 and FLK1, respectively (Fig. S2). Similarly, histological sections revealed that Wnt1/YFP^+^ cells in the perivascular lining of two-day-old mice expressed α-SMA, but not CD31 (Fig. 1D′–D′″). In 2-day-old mice, Mesp1/YFP^+^ dental mesenchymal cells were located in the inner layer of blood vessels and expressed CD31, but not α*-*smooth muscle actin (α*-*SMA)(Fig. 1E′–E′″). Thus, NC-derived and mesoderm-derived cells may contribute to α-SMA^+^ perivascular cells and CD31^+^ endothelial cells, respectively.

**Figure 2:**
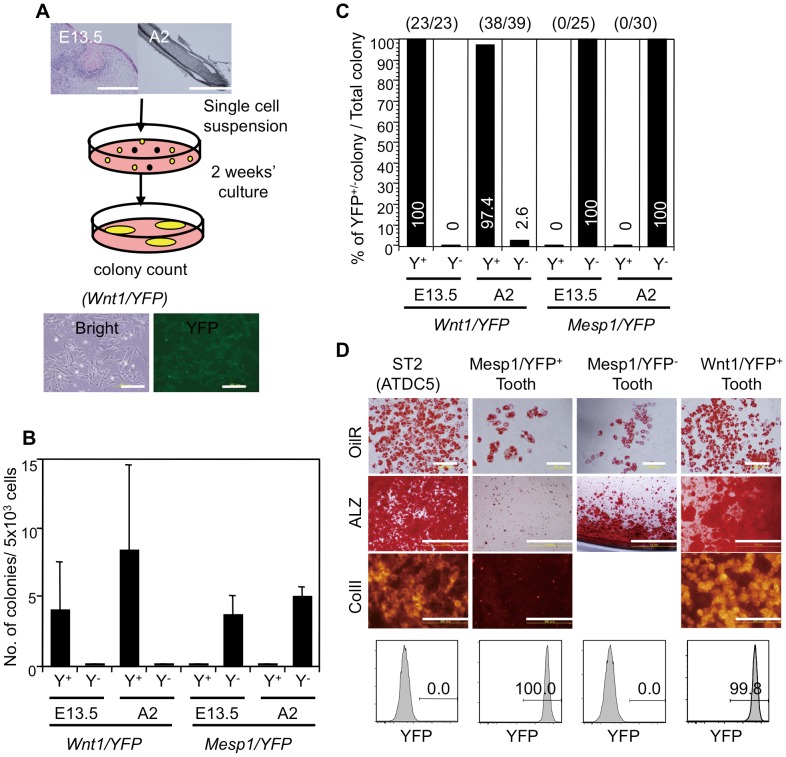
CFU-F assays and differential potential of dental mesenchymal cells of *Wnt1-Cre/YFP* and *Mesp1-Cre/YFP* mice. (A) Protocol of the CFU-F assays and fibroblastic colonies obtained from *Wnt1-Cre/YFP* mice. Scale bars, 200 µm. (B) Numbers of colonies induced from dental mesenchymal cells including both YFP^+^ and YFP^−^ cells of E13.5 and 2-day-old *Wnt1-Cre/YFP* and *Mesp1-Cre/YFP* mice. Values represent the mean (SD) of triplicate cultures of two independent experiments. Y^+^, YFP^+^ colonies; Y^−^, YFP^−^ colonies. (C) Percentages of YFP^+^ and YFP^−^ colonies induced from dental mesenchymal cells of *Wnt1-Cre/YFP* and *Mesp1-Cre/YFP* mice. Figures within parentheses indicate the number of YFP^+^ colonies of the total number of colonies. Values represent percentages and colony numbers of triplicate cultures of two independent experiments (*n* = 6/group). (D) Differentiation potential of NC-derived and mesoderm-derived dental mesenchymal cells into adipocytes, osteoblasts, and chondrocytes. Cells from YFP^+^ or YFP^−^ colonies using dental mesenchymal cells from *Wnt1-Cre/YFP* or *Mesp1-Cre/YFP* mice were collected using a cell sorter. YFP expression in each cell preparation is shown in the lower panel. The cultured cells were stained with oil red O (OilR), alizarin red (ALZ), and anti-type II collagen (Col II) antibody to detect adipocytes, osteoblasts, and chondrocytes, respectively. ST2 and ATDC5 cells were the positive controls. The experiments were repeated twice and one representative experiment is presented. Scale bars, 200 µm in OilR and Col II and 1 mm in ALZ.

We performed CFU-F assays to determine the origin of dental MSCs, which are functional assays for measuring MSCs *in vitro* (Fig. 2A). We used unfractionated cells, including YFP^+^ and YFP^−^ cells from E13.5 *Wnt1/YFP* or *Mesp1*/*YFP* embryos, but all colonies comprised Wnt1/YFP^+^ or Mesp1/YFP^−^cells (Fig. 2B, C). Using unfractionated dental pulp cells from two-day-old mice, we found that all colonies were Wnt1/YFP^+^, except one, and that all consisted of Mesp1/YFP^−^ cells (Fig. 2C). Four-week-old *Mesp1/YFP* and *Wnt1/YFP* mice yielded similar results (Table S1).

To estimate the self-renewal activity of CFU-Fs, we examined the capacity for repeatable colony formation (secondary or tertiary CFU-F assays). Cells from primary colonies were used to detect secondary CFU-Fs. The frequency of secondary colony formation (0.37%–2.00%) was approximately 10 times higher than that of primary colony formation (0.06%–0.29%) (Table S1). These results suggest that dental CFU-Fs contain self-renewing MSCs. All secondary colonies were Wnt1/YFP^+^, but only one secondary colony from four-week-old *Mesp1/YFP* mice was composed of Mesp1/YFP^+^ cells in one of two independent experiments (Table S1, Exp. 1). The YFP^+^ cells from *Mesp1/YFP* mice exhibited proliferative capacity. However, the frequency of Mesp1/YFP^+^ colony formation was very low in the tertiary CFU-F assays (0.1%; 2/2,000 cells) compared with Wnt1/YFP^+^ colonies (Fig. S3A). Mesp1/YFP^+^ cells recovered from primary or secondary colonies expressed PDGFRβ, but scarcely expressed CD31, which was similar to Wnt1/YFP^+^ cells (Fig. S3B, C). Thus, CFU-Fs containing dental MSCs in the dental mesenchyme were generated mainly from NC-derived cells and rarely from mesoderm-derived cells.

### Dental CFU-Fs with the potential for differentiation into osteoblasts, adipocytes, and chondrocytes are derived from NC

CFU-Fs are defined as cells with the potential for differentiation into osteoblasts, adipocytes, and chondrocytes *in vitro*. To assess whether colonies induced from dental mesenchymal cells could differentiate into these types, we performed CFU-F assays using two-day-old or four-week-old *Wnt1/YFP* and *Mesp1/YFP* mice. Cell suspensions prepared from YFP^+^ colonies were cultured using reagents to induce their differentiation. Because Mesp1/YFP^+^ dental mesenchymal cells formed few colonies in the CFU-F assays, Mesp1/YFP^+^ cells were sorted and cultured. The cultured cells were stained with ALZ, OilR, or anti-type II collagen (Col II) antibody after 2–3 weeks to detect osteoblasts, adipocytes, and chondrocytes, respectively. Large numbers of ALZ^+^, OilR^+^, and Col II^+^ cells were induced from Wnt1/YFP^+^ dental mesenchymal cells (Fig. 2D). P0/YFP^+^ dental mesenchymal cells also produced similar results. However, OilR^+^ cells and a small number of ALZ^+^ cells, but no Col II^+^ cells, were induced from Mesp1/YFP^+^ dental mesenchymal cells. In contrast, large numbers of ALZ^+^ and OilR^+^ cells were induced from Mesp1/YFP^−^ cells (Fig. 2D). These results indicate that dental CFU-Fs with MSC properties were present in the dental pulp and were derived only from NC.

### Roles of PDGFRs in CFU-Fs from dental mesenchymal cells

**Figure 3:**
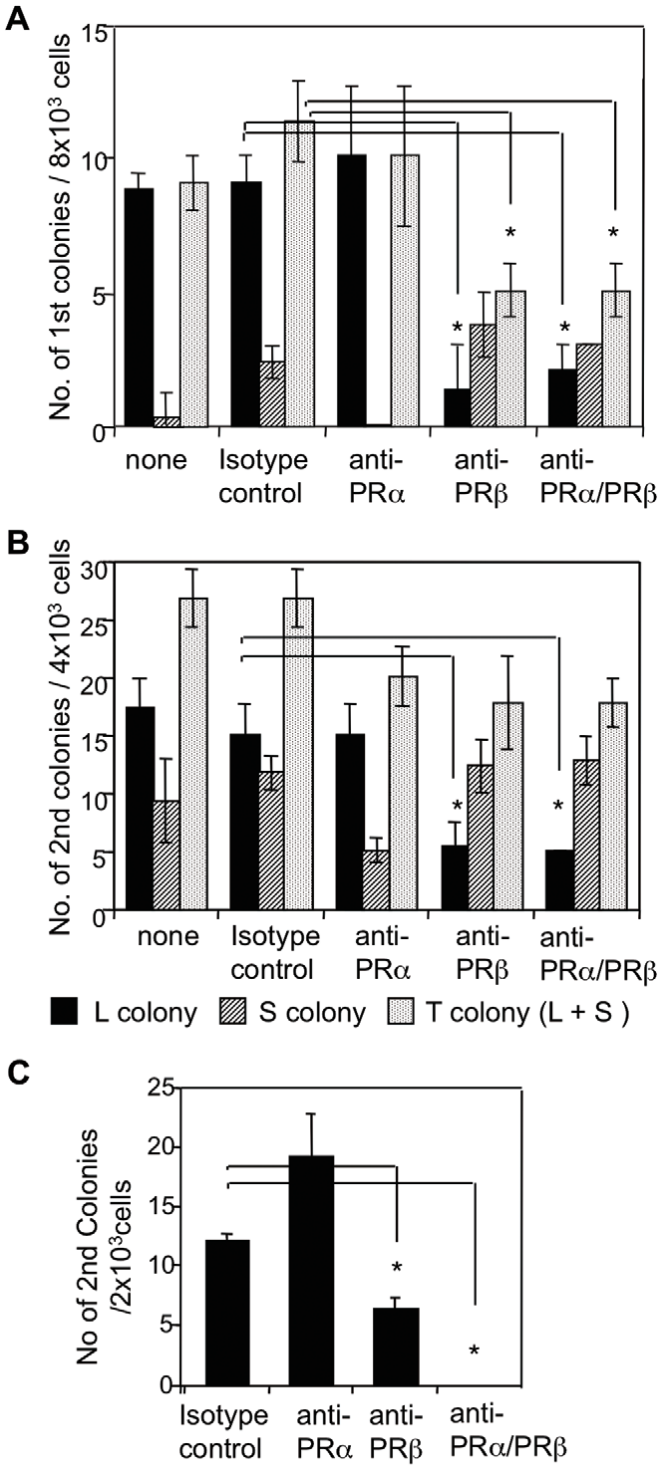
Effects of inhibitory antibodies against PDGFRα and/or PDGFRβ in CFU-F assays using dental mesenchymal cells. (A) Numbers of primary colonies (1st CFU-F) induced from dental mesenchymal cells from 4-week-old mice in the presence of anti-PDGFRα (APA5) and/or anti-PDGFRβ (APB5). None, no antibody. Numbers of large (L, >50 cells), small (clusters) (S, 8–50 cells), and total colonies (T, L+S colonies) are shown. (B) Numbers of secondary colonies (2nd CFU-F) induced from primary colonies of 4-week-old mice in the presence of anti-PDGFRα and/or anti-PDGFRβ. The primary CFU-F assay was performed in the absence of these antibodies. Numbers of large, small, and total colonies are shown. (C) Numbers of secondary colonies (2nd CFU-F) induced from primary colonies from 4,000 dental mesenchymal cells of 4-week-old mice in the presence of anti-PDGFRα and/or anti-PDGFRβ. The secondary CFU-F assay was performed in the absence of antibodies. Values represent the means (SD) of triplicate cultures. Asterisks indicate a significant difference from the number of colonies in the presence of the isotype-matched control antibody (*p*<0.05). The experiments were repeated twice and one representative experiment is presented.

It is known that PDGFRs are expressed on MSCs, and PDGFRα^+^ and PDGFRβ^+^ cells were found among Wnt1/YFP^+^ dental mesenchymal cells. PDGF is related to the CFU-F colony size when culturing BM cells in serum-deprived conditions [33], but the roles of PDGFRs in dental mesenchymal cells are unclear. We assessed their roles in colony formation using inhibitory antibodies against PDGFRα (anti-PDGFRα) and/or PDGFRβ (anti-PDGFRβ). We classified the colonies as either large (>50 cells) or small (approximately 8–50 cells). Anti-PDGFRα alone had little effect on colony formation, whereas anti-PDGFRβ decreased the number of large colonies to 15% of that observed in the presence of the isotype control or anti-PDGFRα in the primary CFU-F assay (Table S2, Fig. 3A). The total colony number observed in the presence of anti-PDGFRβ was 55% of that observed in the presence of anti-PDGFRα or the isotype control (Table S2, Fig. 3A). Similarly, in the secondary CFU-F assay, the number of total and large colonies formed in the presence of anti-PDGFRβ were 65% and 33%, respectively, of those observed in the presence of the isotype control (Fig. 3B). Thus, signaling by PDGFRβ is important for maintaining dental CFU-Fs. However, we cannot rule out the possibility that PDGFRβ signaling promoted the proliferation of CFU-F descendants (Fig. 3A, B), because anti-PDGFRβ affected the number of large colonies rather than the total number of colonies. To clarify this issue, we performed a primary CFU-F assay in the presence of anti-PDGFRβand/or anti-PDGFRα, and a secondary CFU-F assay in the absence of these antibodies. Cells prepared from colonies in the primary CFU-F assay treated only with anti-PDGFRβ or with both anti-PDGFRα and anti-PDGFRβ produced 50% or 0% of cells prepared from colonies observed in the presence of the isotype control in the secondary CFU-F assay (Fig. 3C). Thus, PDGFRβ signaling is important for maintaining self-renewal of dental CFU-Fs, rather than proliferation of CFU-F descendants.

### Origins and characteristics of thymic and BM mesenchymal cells

Previously, we reported that multipotent NC-derived cells participate in the formation of fetal thymic and dental mesenchyme [31], [32]. However, the origins and characteristics of BM and thymic mesenchymal cells and their MSCs remain unclear. Thus, we examined whether NC-derived and mesoderm-derived cells contributed to BM and thymic mesenchyme from the fetal stage to the adult stage.

**Figure 4:**
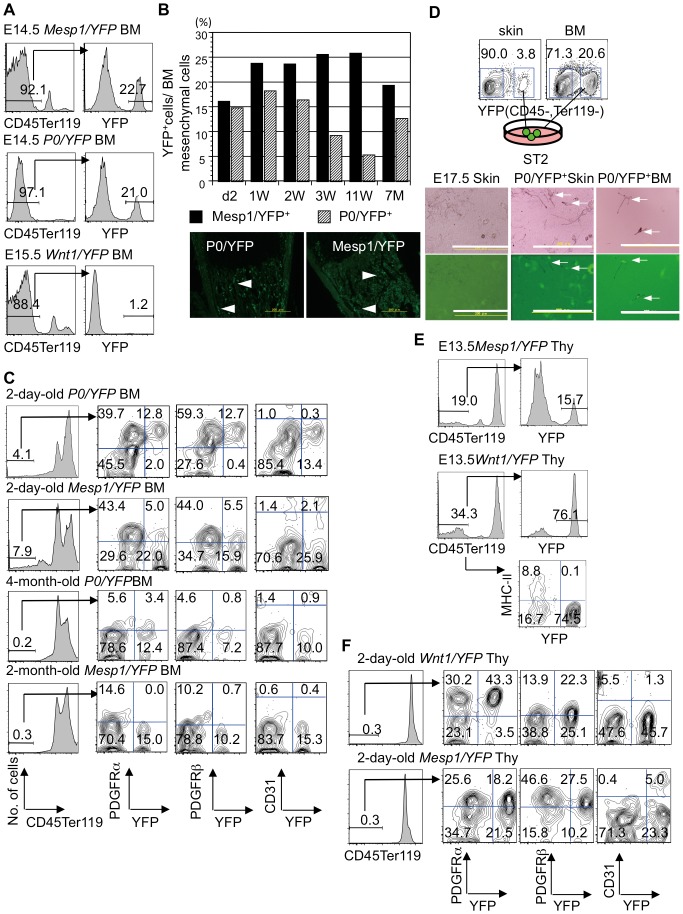
Origins and characteristics of mesenchymal cells in BM and thymus. (A) Contributions of YFP^+^ mesenchymal cells from *Mesp1-Cre/YFP*, *P0-Cre/YFP*, and *Wnt1-Cre/YFP* embryos to BM. (B) YFP^+^ cells from BM mesenchyme of *P0-Cre/YFP* and *Mesp1-Cre/YFP* mice from neonatal to adult stages. The mean of two independent experiments is shown. W, week; M, month. Immunohistochemistry for YFP in BM of 2-day-old *P0-Cre/YFP* and *Mesp1-Cre/YFP* mice (lower panel). White arrowheads indicate YFP^+^ cells in the trabecular and cortical bone. (C) Expression of YFP, PDGFRα, PDGFRβ, and CD31 on BM mesenchymal cells prepared from *P0-Cre/YFP*, *Wnt1-Cre/YFP*, and *Mesp1-Cre/YFP* mice. (D) Induction of melanocytes from the sorted E17.5 P0/YFP^+^ BM (6.5×10^3^). The P0/YFP^+^ cells from the skin of the same mice (P0/YFP^+^ skin) and C57BL/6 mice were the controls. White arrows indicate pigmented and YFP^+^ melanocytes. Scale bars, 200 µm. (E) Contributions of YFP^+^ mesenchymal cells from *Mesp1-Cre/YFP* and *Wnt1-Cre/YFP* embryos to thymus. (F) Expression of YFP, PDGFRα, PDGFRβ, and CD31 on thymic mesenchymal cells prepared from *Wnt1-Cre/YFP*, and *Mesp1-Cre/YFP* mice. The experiments were repeated and one representative experiment is presented.

In the BM, both Mesp1/YFP^+^ and P0/YFP^+^ (Wnt1/YFP^+^) cells contributed to mesenchyme in E14.5 or E15.5 embryos, two-day-old mice, and adult mice (Fig. 4A, B). However, half of the BM mesenchyme was composed of cells other than P0/YFP^+^ (Wnt1/YFP^+^) and Mesp1/YFP^+^ cells (Fig. 4A–C). Immunohistochemistry detected two-day-old P0/YFP^+^ and Mesp1/YFP^+^ BM mesenchymal cells in trabecular and cortical bone (Fig. 4B). Subsequently we reported that fetal thymic and dental mesenchymal cells have the potential to differentiate into melanocytes (a highly reliable marker of NC-derived cells) [31], [32]. We confirmed the presence of NC-derived cells in BM, by testing whether these mesenchymal cells differentiate into melanocytes. Pigmented melanocytes were induced from YFP^+^ cells in BM and YFP^+^ cells from the skin of E17.5 *P0/YFP* embryos (Fig. 4D). The results suggested that NC-derived cells were present in the BM.

**Figure 5:**
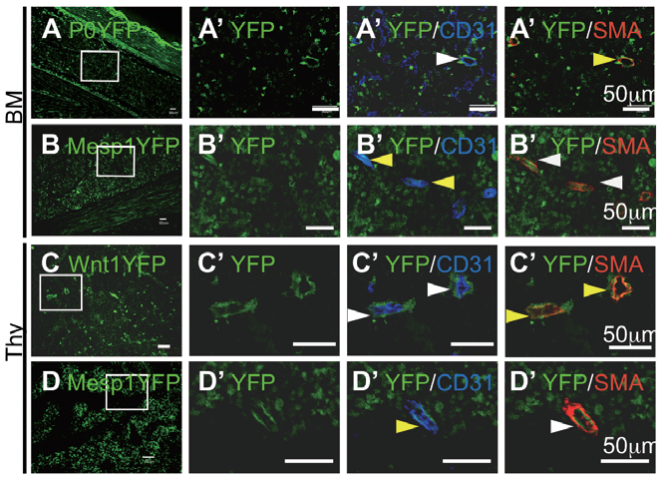
Immunohistochemistry of the BM or thymus of *P0-Cre/YFP*, *Mesp1-Cre/YFP*, and *Wnt1-Cre/YFP* mice. (A–D) Immunohistochemistry for YFP, CD31, and α-SMA in the BM (A, B) or thymus (C, D) of 2-day-old *P0-Cre/YFP* (A), *Mesp1-Cre/YFP* (B, D), and *Wnt1-Cre/YFP* (C) mice. High-magnification views (A′–D′) of the boxed areas in (A–D), respectively. Yellow and white arrowheads indicate positive cells for each antibody to indicate the presence or absence of YFP^+^ cells, respectively. Scale bars, 50 µm. The experiments were repeated and one representative experiment is presented.

Next, we tested the expression of PDGFRs in BM mesenchymal cells. Unlike dental mesenchyme, PDGFRα- and/or PDGFRβ-expressing cells were present in the YFP^+^ and YFP^−^ fractions of BM mesenchymal cells from two-day-old *Mesp1/YFP* and *P0/YFP* mice (Fig. 4 C). The sections showed that two-day-old P0/YFP^+^ BM mesenchymal cells around the blood vessels expressed α*-*SMA (Fig. 5A). Furthermore, Mesp1/YFP^+^ BM mesenchymal cells of the same age expressed CD31 in blood vessels (Fig. 5B). Thus, NC-derived and mesoderm-derived cells in the BM may contribute to α-SMA^+^ perivascular cells and CD31^+^ endothelial cells, respectively.

In the thymus, most E13.5 thymic mesenchymal cells (except MHC class II^+^ thymic epithelial cells) consisted of either Wnt1/YFP^+^ or Mesp1/YFP^+^ cells (Fig. 4E), while 86% of CD45^−^Ter119^−^ thymic mesenchymal cells from two-day-old mice were composed of either Wnt1/YFP^+^ cells or Mesp1/YFP^+^ cells (Fig. 4F). Similar to BM, PDGFRα- and/or PDGFRβ-expressing cells were observed in the YFP^+^ and YFP^−^ fractions of thymic mesenchymal cells from two-day-old *Mesp1/YFP* and *Wnt1/YFP* mice (Fig. 4F). The sections indicated that two-day-old Wnt1/YFP^+^ thymic mesenchymal cells around the blood vessels expressed α*-*SMA, whereas Mesp1/YFP^+^ thymic mesenchymal cells of the same age expressed CD31 in blood vessels (Fig. 5C, D). Thus, NC-derived and mesoderm-derived cells in the thymus may contribute to α-SMA^+^ perivascular cells and CD31^+^ endothelial cells, respectively.

### Origins of BM and thymic CFU-Fs and roles of PDGFRs in these CFU-Fs

**Figure 6:**
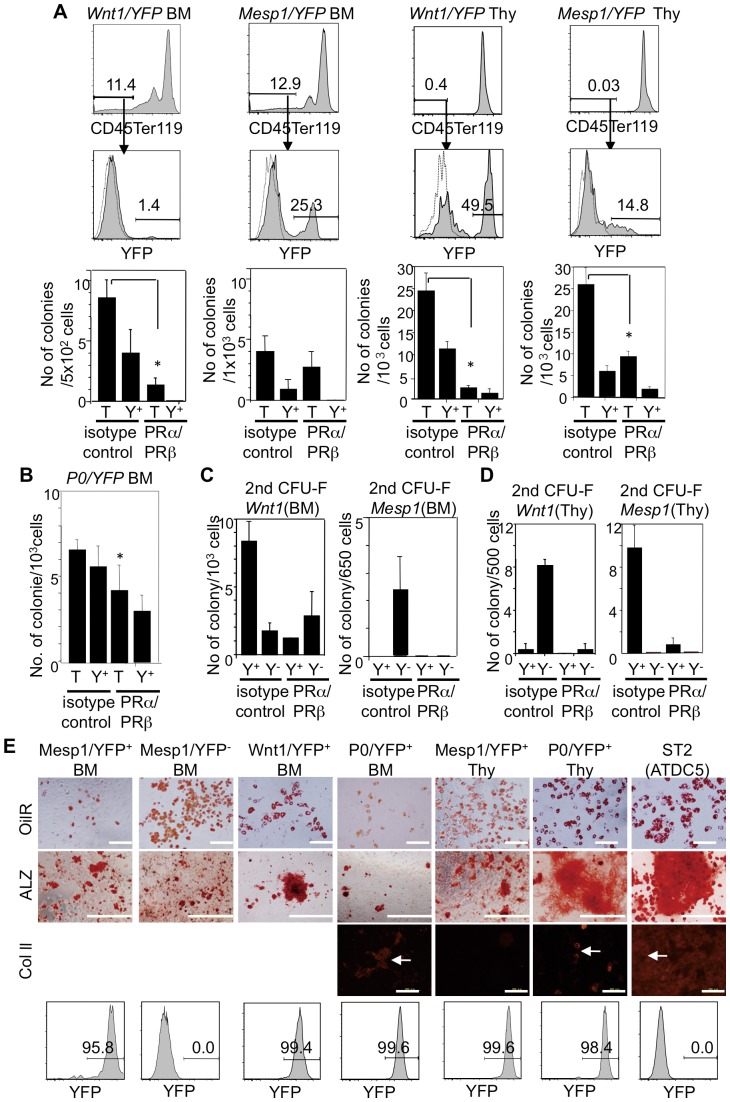
Origins and characteristics of BM and thymic CFU-Fs and roles of PDGFRs in these CFU-Fs. (A) Contributions of YFP^+^ mesenchymal cells (CD45-Ter119-cells) from 2-day-old *Wnt1-Cre/YFP* and *Mesp1-Cre/YFP* mice to BM and thymus. BM and thymic CFU-F assays were performed in the presence of anti-PDGFRβ (APB5) and anti-PDGFRα (APA5) or isotype-matched control antibody. (B) Numbers of colonies induced from BM mesenchymal cells from 2-day-old *P0-Cre/YFP* mice in the presence of the anti-PDGFRβ and anti-PDGFRα. (C, D) The secondary CFU-F assay using cells from primary colonies induced from 1,000 BM (C) and thymic (D) mesenchymal cells of 2-day-old *Wnt1-Cre/YFP* and *Mesp1-Cre/YFP* mice in the presence of anti-PDGFRα and anti-PDGFRβ antibodies. The secondary CFU-F assay was performed in the absence of antibodies. T, total colonies; Y^+^, YFP^+^ colonies; Y^−^, YFP^−^ colonies. Values represent the mean (SD) of triplicate cultures. Asterisks indicate a significant difference in the number of colonies compared with that of the isotype-matched control antibody (*p*<0.05). (E) Differentiation potential of thymic and BM mesenchymal cells from *Wnt1-Cre/YFP*, *P0-Cre/YFP*, and *Mesp1-Cre/YFP* mice into adipocytes, osteoblasts, and chondrocytes. YFP expression in each cell preparation is shown in the lower panel. The cultured cells were stained with oil red O (OilR), alizarin red (ALZ), and anti-type II collagen (Col II) antibody to detect adipocytes, osteoblasts, and chondrocytes, respectively. ST2 and ATDC5 cells were the positive controls. The experiments were repeated twice and one representative experiment is presented. Scale bars, 200 µm in OilR and Col II in (E), and 1 mm in (ALZ in E). White arrows indicate positive cells against Col II antibody.

The observed discrepancy between PDGFR-expressing cells in the dental mesenchyme, the thymus, and the BM mesenchyme necessitated an assessment of CFU-F origins in the thymus and BM mesenchyme. We performed CFU-F assays using BM mesenchymal cells from two-day-old *Wnt1/YFP* (*P0/YFP*) and *Mesp1/YFP* mice. Unlike the dental mesenchymal cells, Mesp1/YFP^+^ and Wnt1/YFP^+^ (P0/YFP^+^) BM mesenchymal cells exhibited colony-forming capacity (Fig. 6A). Unlike dental and thymic mesenchymal cells, it was unclear whether Mesp1/YFP^−^ cells were Wnt1/YFP^+^ (P0/YFP^+^) NC-derived cells or whether Wnt1/YFP^−^ (P0/YFP^−^) cells were Mesp1/YFP^+^ mesoderm-derived cells, because the mesenchymal cells comprised 1% Wnt1/YFP^+^, 15% P0/YFP^+^, and 27% Mesp1/YFP^+^ cells, indicating that 57% of cells were not Wnt1/YFP^+^ (P0/YFP^+^) NC-derived cells or Mesp1/YFP^+^ mesoderm-derived cells. Therefore, Wnt1/YFP^−^ (P0/YFP^−^) and Mesp1/YFP^−^ mesoderm-derived cells may be present in BM (Figs. 4C, 6A), Nevertheless, Wnt1/YFP^+^ and P0/YFP^+^ cells comprised 44% and 85% of BM CFU-Fs, respectively (Fig. 6A, B). P0/YFP^+^ BM mesenchymal cells from seven-month-old mice continued to exhibit a colony-forming capacity, and cells prepared from these colonies expressed PDGFRα and PDGFRβ (Fig. S4).

A secondary CFU-F assay was performed to investigate the self-renewal activity of BM CFU-Fs. Secondary CFU-Fs in the BM comprised 80% Wnt1/YFP^+^ and 20% Wnt1/YFP^−^ cells, or 100% Mesp1/YFP^−^ cells (Fig. 6C, D). These results suggest that BM MSCs are derived mainly from NC and that they are maintained in older mice.

BM CFU-Fs were present in PDGFRα^+^ and PDGFRβ^+^ cells. Because the role of PDGFRs in BM CFU-Fs remained unclear; therefore, we examined the effects of anti-PDGFR antibodies on colony formation. Similar to dental CFU-Fs, the number of CFU-Fs induced from BM cells decreased with the addition of anti-PDGFRα and anti-PDGFRβ to the culture (Fig. 6A, B). The number of YFP^+^ and YFP^−^ colonies generated from BM mesenchymal cells of *P0/YFP*, *Wnt1/YFP* and *Mesp1/YFP* mice decreased (Fig. 6A, B). To assess the effects of these antibodies on the self-renewal capacity of CFU-Fs, we performed a primary CFU-F assay with anti-PDGFRβ and anti-PDGFRα antibodies, and a secondary CFU-F assay was performed without either antibody. In the BM mesenchymal cells from *Wnt1/YFP* mice, we found that the number of secondary colonies treated with both antibodies was 40% of that observed in the presence of the isotype control (Fig. 6C). The number of YFP^+^ secondary colonies induced from the BM of *Wnt1/YFP* and *Mesp1/YFP* mice were reduced in the presence of both antibodies Fig. 6C, D). Irrespective of the origins of cells, it was clear that PDGFRβ signaling was important for maintaining self-renewal of BM CFU-Fs.

Next, we performed CFU-F assays using thymic mesenchymal cells from two-day-old *Wnt1/YFP* and *Mesp1/YFP* mice. Mesp1/YFP^+^ and Wnt1/YFP^−^ thymic mesenchymal cells exhibited colony-forming capacity (Fig. 6A). In particular, secondary CFU-Fs in the thymus were derived almost entirely from Mesp1/YFP^+^ or Wnt1/YFP^−^ cells (Fig. 6C, D). Similar to the BM CFU-Fs, the number of CFU-Fs induced from thymic cells decreased with the addition of anti-PDGFRα and anti-PDGFRβ to the culture (Fig. 6A, B). The number of YFP^+^ and YFP^−^ colonies generated using thymic mesenchymal cells from *Wnt1/YFP* and *Mesp1/YFP* mice decreased (Fig. 6A, B). Furthermore, thymic mesenchymal cells from *Wnt1/YFP* mice and *Mesp1/YFP* mice treated with anti-PDGFRα and anti-PDGFRβ antibody exhibited an unusual capacity for secondary colony formation (Fig. 6C, D). Similar to the BM, PDGFRβ signaling is important for maintaining thymic CFU-Fs containing self-renewal MSCs, irrespective of their origin.

### BM and thymic mesenchymal cells can differentiate into osteoblasts, adipocytes, and chondrocytes

We cultured CFU-F progeny to assess the differentiation potential of BM and thymic mesenchymal cells and then found that CFU-F progeny from Wnt1/YFP^+^(P0/YFP^+^) BM and thymic mesenchymal cells differentiated into osteoblasts, adipocytes, and chondrocytes (Fig. 6E). Unlike dental mesenchymal cells, Mesp1/YFP^+^ BM and thymic mesenchymal cells had the potential to differentiate into osteoblasts and adipocytes (Fig. 6E). Thus, CFU-Fs with MSC properties develop mainly from NC and the mesoderm in the BM and thymic mesenchyme.

### Dental mesenchymal cells support B lymphopoiesis and osteoclastogenesis

**Figure 7:**
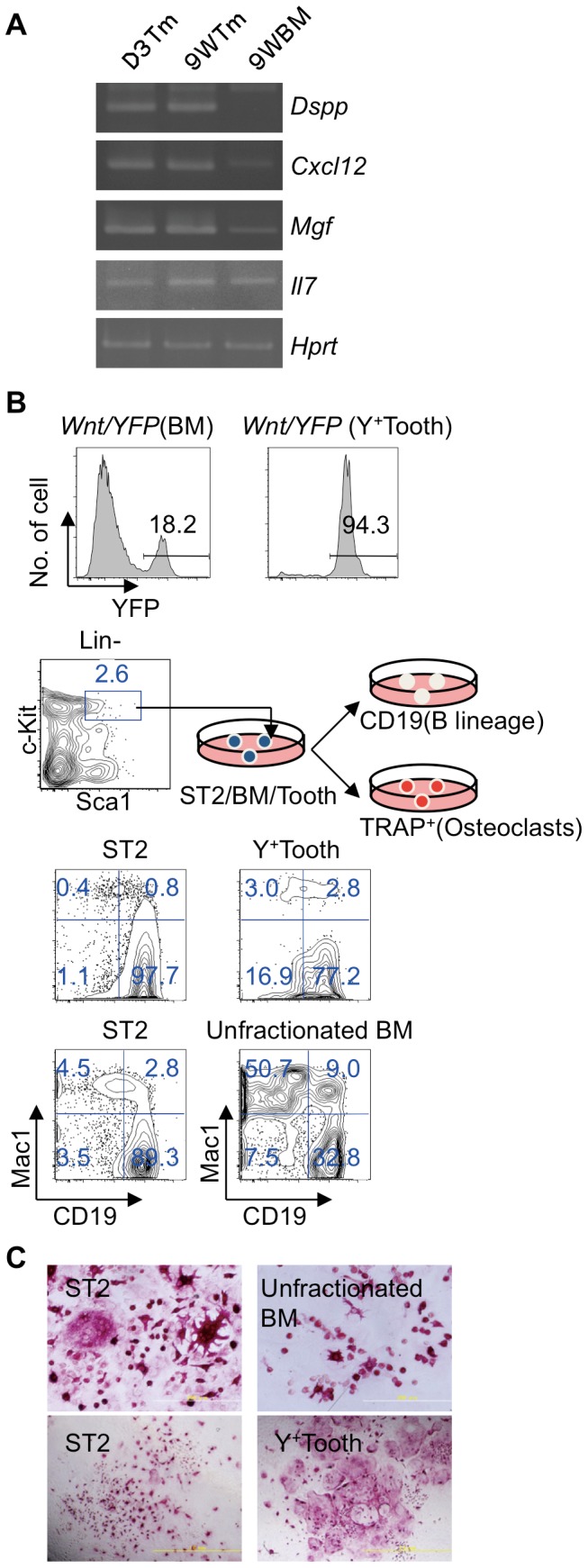
NC-derived dental mesenchymal cells support B lymphopoiesis and osteoclastogenesis. (A) Expression of genes encoding hematopoietic factors on CD45^−^ and Ter119^−^ BM mesenchymal cells and dental mesenchymal cells. RT-PCR was performed using RNA from these cells. Hypoxanthine guanidine phosphoribosyl transferase (*Hprt*) was the positive control. D3Tm, dental mesenchymal cells from 3-day-old mice; 9WTm, dental mesenchymal cells from 9-week-old mice; 9WBM, CD45^−^ and Ter119^−^ BM mesenchymal cells from 9-week-old mice. (B) Induction of B lineage cells. Unfractionated BM and purified YFP^+^ dental mesenchymal cells (Y^+^ Tooth) from 3-day-old *Wnt1-Cre/YFP* mice were cultured. Percentages of YFP^+^ cells in the CD45^−^ and Ter119^−^ fractions after 2 weeks' culture (upper panels). BM-derived 200 KSL cells were then cultured on purified YFP^+^ dental mesenchymal cells (Y^+^ Tooth) or unfractionated BM mesenchymal cells in the presence of rmIL-7. Mac1 and CD19 were used as Myeloid cell lineage and B cell lineage markers. (C) Osteoclast induction. One hundred KSL cells were cultured on unfractionated BM and YFP^+^ dental mesenchymal cells (Y^+^ Tooth) prepared from the same mice in the presence of 1α,25(OH)_2_D_3_, and DEX. TRAP staining was performed to detect osteoclasts. ST2 stromal cells were used as the positive control to support the differentiation of B lymphocytes and osteoclasts. Each experiment with BM or dental mesenchyme is shown with ST2 as the control (B, C). Each experiment was repeated twice and one representative experiment is presented. Scale bars, 200 µm.

Dental and BM mesenchymal cells have many similarities. BM mesenchymal cells support the differentiation of B lymphocytes and osteoclasts [34], [35]. However, it is unclear whether dental mesenchymal cells support the differentiation of these cells. Thus, we prepared dental and BM mesenchymal cells from three-day-old or nine-week-old *Wnt1/YFP* mice. First, we examined the expression of genes encoding the critical hematopoietic factors stem cell factor (SCF), interleukin-7 (IL-7), and CXC chemokine ligand 12 (CXCL12) [36]. Interestingly, dental mesenchyme expresses *Dentin sialophosphoprotein* (*Dspp*, which is an odontoblast-specific gene), *Mgf, Il7*, and *Cxcl12*, as does BM mesenchyme (Fig. 7A). To determine whether dental mesenchyme supported the differentiation of B lymphocytes, we prepared dental and BM mesenchymal cells from *Wnt1/YFP* mice. Two weeks later, 200 c-Kit^+^ Sca1^+^ Lineage^−^ (KSL) cells were isolated from the BM of C57BL/6 mice were cultured in purified YFP^+^ dental mesenchyme, which contained 94% YFP^+^ cells, and unfractionated BM mesenchymal cells, which contained 18% YFP^+^ cells with rmIL-7 (Fig. 7B). Two weeks later, we found that a large number of CD19^+^ cells (B lineage cells) were induced on Wnt/YFP^+^ dental mesenchymal cells (Y^+^Tooth), BM mesenchymal cells (unfractionated BM), and ST2 cells, which support the differentiation of B lymphocytes and osteoclasts (Fig. 7B). Next, we studied the possible effects of Wnt/YFP^+^ dental mesenchymal cells on osteoclast induction by culturing 100 KSL cells on these mesenchymal cells in the presence of 1α,25(OH)_2_D_3_ and Dex. After six days, we induced tartrate-resistant acid phosphatase (TRAP)^+^ multinucleated osteoclasts on Wnt/YFP^+^ dental mesenchymal, BM mesenchymal, and ST2 cells (Fig. 7C). We found that 94% of these dental mesenchymal cells were Wnt/YFP^+^ NC-derived cells, which indicated that NC-derived dental mesenchymal cells had similar properties to BM mesenchymal cells in terms of their support of B lymphopoiesis and osteoclastogenesis.

## Discussion

**Figure 8:**
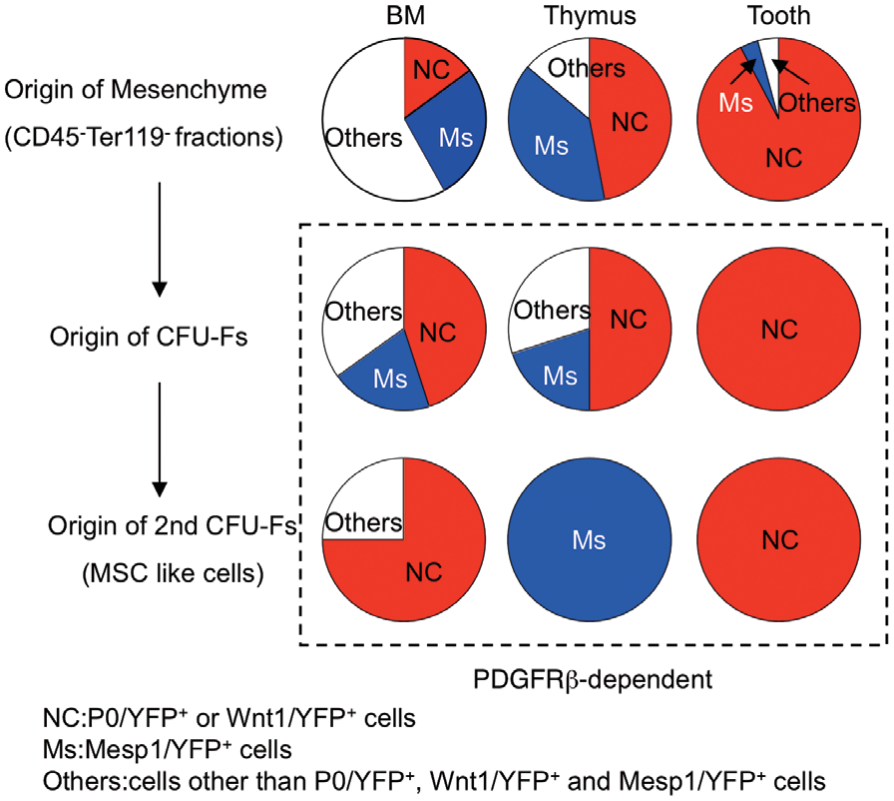
Origin and properties of dental, thymic, and BM mesenchymal cells and their CFU-Fs. Percentages of NC-derived and mesoderm-derived cells comprising BM, thymic, and dental mesenchyme (CD45^−^ and Ter119^−^ cells). YFP^+^ cells in CD45^−^ and Ter119^−^ cells from *P0-Cre/YFP* and *Wnt1-Cre/YFP* mice represent NC-derived cells (NC). YFP^+^ cells in CD45^−^ and Ter119^−^ cells from *Mesp1-Cre/YFP* mice represent mesoderm-derived cells (Ms). YFP^−^ cells other than YFP^+^ cells in the CD45^−^ and Ter119^−^ cells from *P0-Cre/YFP* (*Wnt1-Cre/YFP*) and *Mesp1-Cre/YFP* mice represent others. The expression of primary and secondary CFU-Fs *in vitro* was dramatically affected by PDGFRβ inhibition, irrespective of tissue and origin.

This study aimed to investigate the origin and characteristics of dental, thymic, and BM mesenchymal cells and MSCs. We used *Wnt1/YFP* and *P0/YFP* mice as markers of NC-derived cells and *Mesp1/YFP* mice as markers of mesoderm-derived cells. Most dental and thymic mesenchymal cells are composed of Wnt1/YFP*^+^* NC-derived cells or Mesp1/YFP^+^ mesoderm-derived cells (Fig. 8). However, 57% BM mesenchyme comprises cells other than Wnt1/YFP^+^ (P0/YFP^+^) NC-derived cells or Mesp1/YFP^+^ mesoderm-derived cells (Fig. 8). The lateral plate mesoderm (LPM) generates the limb skeleton [37]. Moreover, the *Mesp1* gene is expressed mainly in LPM-derived head mesenchyme and cardiac mesoderm. Therefore, expression of *Mesp1* gene may be insufficient to allow Cre-mediated recombination in LPM, or possibly other mesoderm-derived cells such as the paraxial mesoderm may contribute to LPM. The origin of BM mesenchymal cells may differ from that of dental and thymic mesenchymal cells.

Approximately similar numbers of P0/YFP^+^ and Wnt1/YFP^+^ cells were observed in the tooth and thymus, whereas larger numbers of P0/YFP^+^ cells than Wnt1/YFP*^+^* cells were detected in BM. Although we cannot explain the difference between the numbers of YFP^+^ cells, the fact that YFP^+^ BM cells of *P0-Cre/Floxed-EGFP* and *Wnt1-Cre/Floxed-EGFP* mice differentiate into peripheral neurons and glia developing from NC [6], along with our observation that P0/YFP^+^ and Wnt1/YFP^+^ BM mesenchymal cells differentiate into melanocytes, suggests that *P0/YFP* and *Wnt1/YFP* mice mark NC-derived cells. Because *Wnt1-Cre* is expressed in early migratory NC [5], [38], whereas *P0-Cre* is expressed in the early stage of NC cells and in the glial subset of NC cells [30], [39], *P0/YFP* may also label other populations of NC-derived cells in addition to the common population of NC-derived cells labeled by both *P0/YFP* and *Wnt1/YFP* mice in BM. Alternatively, the discrepancy between *P0/YFP* and *Wnt1/YFP* mice may be attributed to a Cre-switching efficiency. However, YFP^+^ BM mesenchymal cells in *Wnt1/YFP* demonstrated a marked capacity for colony formation, indicating that NC contributes to BM mesenchyme and its MSC. However, ectopic expression of the *P0-Cre* gene occurs in BM cells, because P0/YFP^+^ cells were partially detected in hematopoietic cells and marked some non-NC–derived cells, including a minor population of endothelial cells in adult BM [40]. On the other hand, YFP^+^ cells in *Sox1-Cre/YFP* mice, which help in tracing neuroepithelial cells, contribute to BM mesenchyme and BM MSCs [7]. The percentage of YFP^+^ cells in BM mesenchyme of *Sox1-Cre/YFP* mice was almost similar to that of YFP^+^ cells from *Wnt1/YFP* mice.

The origins of dental pulp stem cells and stem cells from human exfoliated deciduous teeth (SHEDs) are unclear [16], [19]. Because odontoblasts generally develop from NC, SHEDs that can differentiate into odontoblasts, osteoblasts, and neurons may develop from NC-derived cells. Contrary to our results, Iohara et al. reported that side population (SP) cells of adult porcine dental mesenchymal cells can differentiate into odontoblasts, chondrocytes, adipocytes, neurons, and endothelial cells [41]. These multipotent SP cells express CD31, CD34, FLK1, and CD105 (endothelial markers), but scarcely express α-SMA or NG2 (perivascular cell markers). Because endothelial cells are of mesodermal origin [42], these SP cells may be more immature than NC-derived cells or may comprise both NC-derived and mesoderm-derived cells.

Wnt1/YFP^+^, unlike Mesp1/YFP^+^ dental mesenchymal cells, exhibited marked potential for colony formation in the CFU-F assays. Because dental pulp includes small numbers of Mesp1/YFP^+^ mesoderm-derived cells, we calculated the ratio of the number of YFP^+^ colonies to the total number of YFP^+^ mesenchymal cells in the CFU-F assays. The frequency of CFU-Fs within YFP^+^ dental mesenchymal cells was lower than 1/2,040 (1/1,080) and 1/1,178 (1/728) in E13 (2-day-old) *Mesp1/YFP* and *Wnt1/YFP* mice, respectively. Mesp1/YFP^+^ dental mesenchymal cells may rarely possess colony-forming capacity in the CFU-F assays. Although Mesp1/YFP^+^ thymic and BM mesenchymal cells differentiate into adipocytes and osteoblasts, Mesp1/YFP^+^ dental mesenchymal cells rarely differentiate into osteoblasts and chondrocytes *in vitro*, even when they successfully proliferate. Mesp1/YFP^+^ mesoderm-derived cells in the dental mesenchyme may already have lost the potential to differentiate into osteoblasts and chondrocytes or be committed to endothelial cells.

Human dental pulp stem cells or SHEDs are similar to BM MSCs with regard to the expression of cell-surface antigens such as STRO1^+^ and α-SMA^+^ (perivascular cell markers) and CD146^+^ (a perivascular cell and endothelial cell marker) [43]. In mouse dental pulp and BM cells, PDGFR-expressing cells have shown colony-forming capacity *in vitro*. However, unlike dental CFU-Fs, BM and thymic CFU-Fs comprise both Wnt1/YFP^+^ (P0/YFP^+^) NC-derived cells and Mesp1/YFP^+^ mesoderm-derived cells. Furthermore, self-renewing CFU-Fs, including MSCs, consist entirely of Wnt1/YFP^+^ (P0/YFP^+^) NC-derived cells in the teeth, and mostly Wnt1/YFP^+^ (P0/YFP^+^) NC-derived cells in BM. We also showed that NC-derived dental mesenchymal cells expressed genes encoding critical hematopoietic factors such as IL-7, SCF, and CXCL12, and they supported the differentiation of B lymphocytes and osteoclasts [36], [44]. Although it is unclear whether NC-derived and/or mesoderm-derived BM mesenchymal cells support B lymhopoiesis and osteoclasts, it is well known that cotransplantation of BM mesenchymal cells with hematopoietic cells promotes the reconstitution of hematopoiesis [45]. Because dental mesenchymal cells facilitate the easy preparation of MSCs compared with BM stromal cells, dental mesenchyme may represent a useful resource in improving hematopoiesis in patients with hematopoietic disorders. However, it is still not clear the role of NC-derived cells or mesoderm-derived cells in the BM and thymic lympho-hematopoiesis. Further examination is desire to elucidate these roles.

We first indicated that an inhibitory antibody against PDGFRβ decreased the CFU-F count. Retention of 15% CFU-Fs generated in the control culture in the presence of anti-PDGFRβ implies that both PDGFRβ-dependent and PDGFRβ-independent CFU-Fs are present in the dental pulp, BM, and thymus. Simultaneous addition of anti-PDGFRα and anti-PDGFRβ more effectively blocked secondary colony formation in dental and thymic CFU-F progeny. Because PDGFRα is upregulated in response to *Pdgfrb* mutation [46], anti-PDGFRβ–treated cells may increase PDGFRα expression and become sensitive to anti-PDGFRα.

### Conclusion

This is the first report to demonstrate that both NC-derived and mesoderm-derived cells with CFU-F capacity contribute to the dental pulp, thymus, and BM from the fetal stage to the adult stage. Although the origin of self-renewing CFU-Fs differs by tissue, these CFU-Fs are dependent on PDGFRβ irrespective of their origin.

## Materials and Methods

### Animals and Ethics Statement

*P0-Cre*, *Wnt1-Cre*, and *Rosa26EYFP* mice were provided by Drs. K. Yamamura (Kumamoto University), H. Sucov (Southern California University), and H. Enomoto (RIKEN Kobe), respectively [47]. *Mesp1-Cre* and C57BL/6 mice were obtained from the Riken Bioresource Center and Clea Japan, Inc., respectively. All mice were maintained at the Institute of Laboratory Animals, Mie University; all experimental procedures were approved by the Institutional Animal Care and Use Committee of Mie University (approval number 20–22), and were performed according to the Mie University guidelines for laboratory animals.

### Preparation of single-cell suspensions

The mandibular molar tooth buds and dental pulp of the lower incisors were incubated with 2.4 U dispase II (Roche) in 10% FBS/HBSS for 30 min at 4°C and 1 mg/mL collagenase D (Roche) in 10% FBS/HBSS for 2 h at 37°C. Thymi were incubated with 1 mg/mL collagenase D in 10% FBS/HBSS for 1 h at 37°C. Femora and tibia were minced and were incubated with 2.0 U dispase II and 0.1 mg/mL collagenase D in 10% FBS/HBSS for 1 h at 37°C, and then in 2% FBS/HBSS for 1 h at 37°C. The dorsal regions of E9.5 embryos were dissected and incubated with 2.4 U dispase II in 10% FBS/HBSS at 4°C for 1 h, minced, and then incubated with 1 mg/mL collagenase D in 10% FBS/HBSS for 1 h at 37°C. Pipetted single-cell suspensions were used for further procedures.

### RNA isolation and RT-PCR

Total RNA was prepared using Trizol (Invitrogen). cDNA synthesis was carried out using reverse transcriptase (ReverTraAce; Toyobo) and oligo (dT) primers (Toyobo). PCR using cDNA was performed with r*Taq* polymerase (Toyobo) and the forward primers and reverse primers were as follows: *AP2*: 5′-AGGGACTTTGGGTACGTGTG-3′, 5′-AGGGCCTGGGTGAGATAGTT-3′; *p75(NGFR)*: 5′-TGCTGCTGCTGCTGCTGCTGCTTCT-3′, 5′-CGGGTCCACGTGGTTGGCTTCATCT-3′; *Sox10*: 5′-CACTACACCGACCAGCCGTCCACTT-3′, 5′-GATAGAGTCGTATATACTGGCTGCT-3′; *Krox20*: 5′-ACCCCTGGATCTCCCGTATCCGAGT-3′, 5′-GGACAGGGAAACGGCTTTCGATCTG-3′; *Brachyury(T)*: 5′-CTCCAACCTATGCGGACAAT-3′, 5′-CCCCTTCATACATCGGAGAA-3′; *Dentin sialophosphoprotein(Dspp)*: 5′-ACATTGTTGAAAACTCTGTGGCTGTGCCTC-3′, 5′-CATTTGCTGTGCTGTTCTCTCCTCTCGCAT-3′; *Il7*: 5′-ACATCATCTGAGTGCCACA-3′, 5′-CTCTCAGTAGTCTCTTTAG-3′; *Mgf*: 5′-GTGGCAAATCTTCCAAATGA-3′, 5′-CTCGGGACCTAATGTTGAAG-3′; *Cxcl12*: 5′-GCTCTGCATCAGTGACGGTAAAC-3′, 5′-GCAATATCGTACCATATGCTATGGC-3′; *Hprt*: 5′-AGTTCTTTGCTGACCTGCTG-3′, 5′-GCTTTGTATTGGGCTTTTCC-3′. PCR was performed as follows: 94°C for 4 min; 35 cycles at 93°C for 1 min, 55°C (*Mgf*, *Cxcl12*), 56°C (*Il7*), 58°C (*Sox10*, *Dspp*, *Hprt*), 60°C (*AP2*, *Krox20*, *Brachyury*), 63°C (*p75*) for 1 min, and 72°C for 1 min; and extension at 72°C for 7 min.

### Antibodies

The following antibodies were used: biotin-conjugated PDGFRβ (APB5; eBioscience); Pacific Blue-conjugated streptavidin (eBioscience); APC-conjugated PDGFRα (APA5; eBioscience), PDGFRβ (APB5), CD31 (PECAM1) (MEC13.3; eBioscience), Mac1 (M1/70; Biolegend), and c-Kit (2B8; eBioscience); Cy7PE-conjugated CD45 (30-F11; eBioscience), Ter119 (eBioscience), and CD19 (6D5; Biolegend); Pacific Blue-conjugated Sca1 (Ly-6 A/E) (E13-161.7; Biolegend); PE-conjugated MHC Class II (I-Ab) (25-9-17; BD Pharmingen), FLK1 (Avasα1; eBioscience), and CD34 (RAM34; BD Pharmingen); rabbit anti-mouse p75NGFR polyclonal antibody (Chemicon); Alexafluor 405-conjugated goat anti-rabbit IgG (Invitrogen); biotin-conjugated CD4 (GK1.5; eBioscience), CD8α (53-6.7; eBioscience), Mac1 (M1/70), Gr1 (RB6-8C5; eBioscience), B220 (6B2; eBioscience), and Ter119; and PE-conjugated streptavidin.

### Cell sorting and analysis

Cells were analyzed on FACS Aria or FACS Canto II (BD), followed by analyses with FlowJo software (Tree Star).

### Immunohistochemistry

Molar tooth germs, lower incisors, thymi, and femora were fixed in 4% paraformaldehyde and embedded in cryomold for sectioning (10 µm). The following antibodies were used: Alexafluor 488-conjugated rabbit anti-GFP IgG (Invitrogen), rat anti-mouse CD31 (PECAM-1) (MEC13.3), DyLight™ 649-conjugated AffiniPure donkey anti-rat IgG (H+L) (Jackson ImmunoResearch Laboratory), Cy3-conjugated mouse anti–α-smooth muscle actin (α-SMA) (1A4) (Sigma), mouse anti–neuron-specific β-tubulin III (TUJ1; Babco), and Cy3-conjugated AffiniPure donkey anti-mouse polyclonal IgG (HL) (Jackson ImmunoResearch Laboratory). Images were captured using confocal microscopy (Olympus FV1000D).

### CFU-F assays

Mesenchymal cells from tooth buds, dental pulp, BM, and thymi of *P0-Cre/YFP*, *Wnt1-Cre/YFP*, and *Mesp1-Cre/YFP* mice were cultured in α-MEM with 20% FBS in 6-well plates in the presence/absence of inhibitory antibodies against PDGFRα (anti-PDGFRα, APA5) [48] and/or PDGFRβ (anti-PDGFRβ, APB5) [49]; antibodies against c-Kit (ACK4) and IL-7Rα (A7R) were used as isotype-matched controls in BM and thymic cultures, respectively. Inhibitory antibodies against c-Fms (AFS98) were used to inhibit macrophage proliferation. Two weeks later, large (>50 cells) and small colonies (clusters <50 cells) and YFP^+^ and YFP^−^ colonies were scored as primary CFU-Fs [17]. For secondary CFU-F assays to detect self-renewing CFU-Fs, cells from primary colonies were cultured in the presence/absence of antibodies. Selected YFP^+^/YFP^−^ primary colonies were cultured to establish YFP^+^ clones.

### Induction of adipocytes, osteoblasts, and chondrocytes

For osteoblast induction, 100,000 cells were cultured in DMEM medium (Gibco) supplemented with 10% FBS, 10^−7^ M Dexamethasone (DEX; Sigma), 40 nM human ascorbic acid 2 phosphatase (Sigma), 1 nM BMP4 (Neomarker), and 10 mM β-glycerophosphate (Sigma) [32], [50]. For adipocyte induction, 100,000 cells were cultured in α-MEM supplemented with 10% FBS, 0.25 µM DEX, 0.5 mM 3-isobutyl-1-methylxanthine (Sigma), 1 µM Triiodo (Sigma), and 0.2 µ M insulin (Sigma) [51]. For chondrocyte induction, 200,000 cells (1×10^7^/mL) were cultured in α-MEM supplemented with 10% FBS, 10^−7^ M DEX, 40 nM ascorbic acid-2-phosphatase, and 1 nM TGFβ3 or BMP2 (Neomarker) [49]. After 2–3 weeks, cells were stained with oil red-O (OilR), alizarin red (ALZ), and mouse anti-type II collagen antibody (6B3, Neomarker) and Cy3-conjugated goat anti-mouse IgG (Jackson ImmunoResearch Laboratory) to detect adipocytes, osteoblasts, and chondrocytes, respectively. ST2 and ATDC5 cells were the positive controls for osteogenesis and adipogenesis, and chondrogenesis, respectively [52].

### Induction of B lymphocytes and osteoclasts

Dental and BM mesenchymal cells were prepared from 3-day-old *Wnt1-Cre/YFP* mice. Femoral c-Kit^+^ Sca1^+^ Lineage^−^ (KSL) cells were isolated from 8-week-old C57BL/6 mice. For B lymphocyte induction, 200 KSL cells were cultured on purified YFP^+^ dental mesenchymal cells or unfractionated BM mesenchymal cells in RPMI-1640 (Gibco) supplemented with 10% FBS, 5×10^−5^ M 2ME, and 10 ng/mL rmIL-7 (Invitrogen). After 2 weeks, cells collected were analyzed by FACS. For osteoclast induction, 100 KSL cells were cultured on these mesenchymal cells in α-MEM supplemented 10% FBS, 10^−7^ M DEX, and 10^−7^ M 1α, 25-dihydroxyvitamin D (1α,25(OH)_2_D_3_) (Biomol Research Laboratory). After 6 days, TRAP activity of cells was studied to detect osteoclasts [34]. ST2 cells were the positive control used for the differentiation of B lymphocytes and osteoclasts [34], [35].

### Induction of melanocytes from BM mesenchymal cells

Single-cell suspensions from BM of *P0-Cre/YFP* embryos were prepared. Sorted YFP^+^ BM mesenchymal cells from *P0-Cre/YFP* embryos (20,000) were cultured on ST2 cells in α-MEM containing 10% FBS with 10^−7^ M DEX, 20 pM rhbFGF (R&D Systems), 10 pM cholera toxin (Sigma), and 40 nM rhET3 (Peptide Institute) [31]. Skin YFP^+^ cells of the same mice were used as a positive control. The pigmented melanocytes were microscopically examined after 3 weeks.

### Statistical analysis

Data are expressed as means (SD). Statistical significance was assessed using Student's *t*-test.

## Supporting Information

Figure S1 **Expression of NC- and mesoderm-associated genes on cells from** ***P0-Cre/YFP*** **,** ***Wnt1-Cre/YFP*** **, and** ***Mesp1-Cre/YFP*** **mice.** (A) Expression of p75NGFR on cells in the CD45^−^ and Ter119^−^ fractions from E9.5 *P0-Cre/YFP* and *Wnt1-Cre/YFP* embryos. Empty means secondary antibody only (Alexafluor 405-conjugated goat anti-rabbit IgG) without primary antibody. (B) Expression of NC-associated genes on cells from E9.5 *P0-Cre/YFP*, *Wnt1-Cre/YFP*, and *Mesp1-Cre/YFP* embryos (*n* = 4/group). (C) Expression of NC- and mesoderm-associated genes in dental mesenchymal cells from *Wnt1-Cre/YFP* mice (*n* = 4/group). YFP^+^ and YFP^−^ cells were isolated using a cell sorter. RT-PCR was performed using RNA from these cells. Hypoxanthine guanidine phosphoribosyl transferase (*Hprt*) was the positive control; no expression was detected without a template (data not shown).
(TIF)

Figure S2 **Expression of cell-surface antigens related to endothelial cells on dental mesenchymal cells from 4-week-old** ***Wnt1-Cre/YFP*** **and** ***Mesp1-Cre/YFP*** **mice.** Expression of cell-surface antigens related to endothelial cells on YFP^+^ or YFP^−^ dental mesenchymal cells in CD45^−^ and Ter119^−^ fractions from 4-week-old *Wnt1-Cre/YFP* and *Mesp1-Cre/YFP* mice. The experiments were repeated twice and one representative experiment is presented.
(TIF)

Figure S3 **Expression of PDGFR and CD31 on dental mesenchymal cells from CFU-F progenies of 4-week-old** ***Mesp1-Cre/YFP*** **or** ***Wnt1-Cre/YFP*** **mice.** (A) Number of colonies in the tertiary CFU-F assay using YFP^−^ and YFP^+^ dental mesenchymal cells isolated from secondary CFU-F progenies from *Mesp1-Cre/YFP* mice. Values represent the mean (SD) of triplicate cultures. (B) Expression of PDGFRa and PDGFRβ on YFP^−^ and YFP^+^ dental mesenchymal cells from secondary CFU-F progenies from *Mesp1-Cre/YFP* mice. (C) Expression of CD31 and PDGFRβ on YFP^+^ and YFP^−^ cells recovered from primary CFU-F progenies from *Mesp1-Cre/YFP* and *Wnt1-Cre/YFP* mice. The experiments were repeated twice and one representative experiment is presented.
(TIF)

Figure S4 **CFU-Fs of BM mesenchymal cells from 7-month-old** ***P0-Cre/YFP*** **mice.** (A) Numbers of colonies induced from BM mesenchymal cells from 7-month-old *P0-Cre/YFP* mice. (B) Expression of YFP, PDGFRα, and PDGFRβ on cells from these colonies. Values represent the mean (SD) of triplicate cultures. The experiments were repeated twice and one representative experiment is presented.
(TIF)

Table S1 **The number of primary and secondary colonies and the origin of colony-forming cells in dental mesenchymal cells.** Numbers of primary colonies (1st CFU-F, >50 cells) induced from 4×10^3^ dental mesenchymal cells from 4-week-old *Wnt1-Cre/YFP* and *Mesp1-Cre/YFP* mice. Numbers of secondary colonies (2nd CFU-F, >50 cells) induced from 1×10^3^ primary colonies of 4-week-old mice. Values represent the means (SD) of triplicate cultures. Asterisks indicate total number of colonies obtained from triplicate cultures of two independent experiments.
(DOC)

Table S2 **Effects of inhibitory antibodies against PDGFRs in CFU-F assays using dental mesenchymal cells.** Numbers of colonies were induced from 8×10^3^ dental mesenchymal cells prepared from 4-week-old *Wnt1-Cre/YFP* mice in the presence of inhibitory antibody against PDGFRα (APA5) and/or inhibitory antibody against PDGFRβ (APB5). No add means no antibody, and control means isotype-matched control antibody (ACK4). All antibodies were used in 10 mg/ml. Numbers of large (L, >50 cells), small (clusters) (S, <50 cells), and total colonies (T, L+S colonies) are shown. Values represent the means (SD) of triplicate cultures. Asterisks indicate a significant difference from the number of colonies in the presence of the isotype-matched control antibody (*p*<0.05). The experiments were repeated twice and one representative experiment is presented.
(DOC)
