# Supplementary figures and images for: Origins and Properties of Dental, Thymic, and Bone Marrow Mesenchymal Cells and Their Stem Cells
Source: PLoS One. 2012 Nov 21;7(11):e46436. doi: 10.1371/journal.pone.0046436 (PMC3504117; doi:10.1371/journal.pone.0046436)

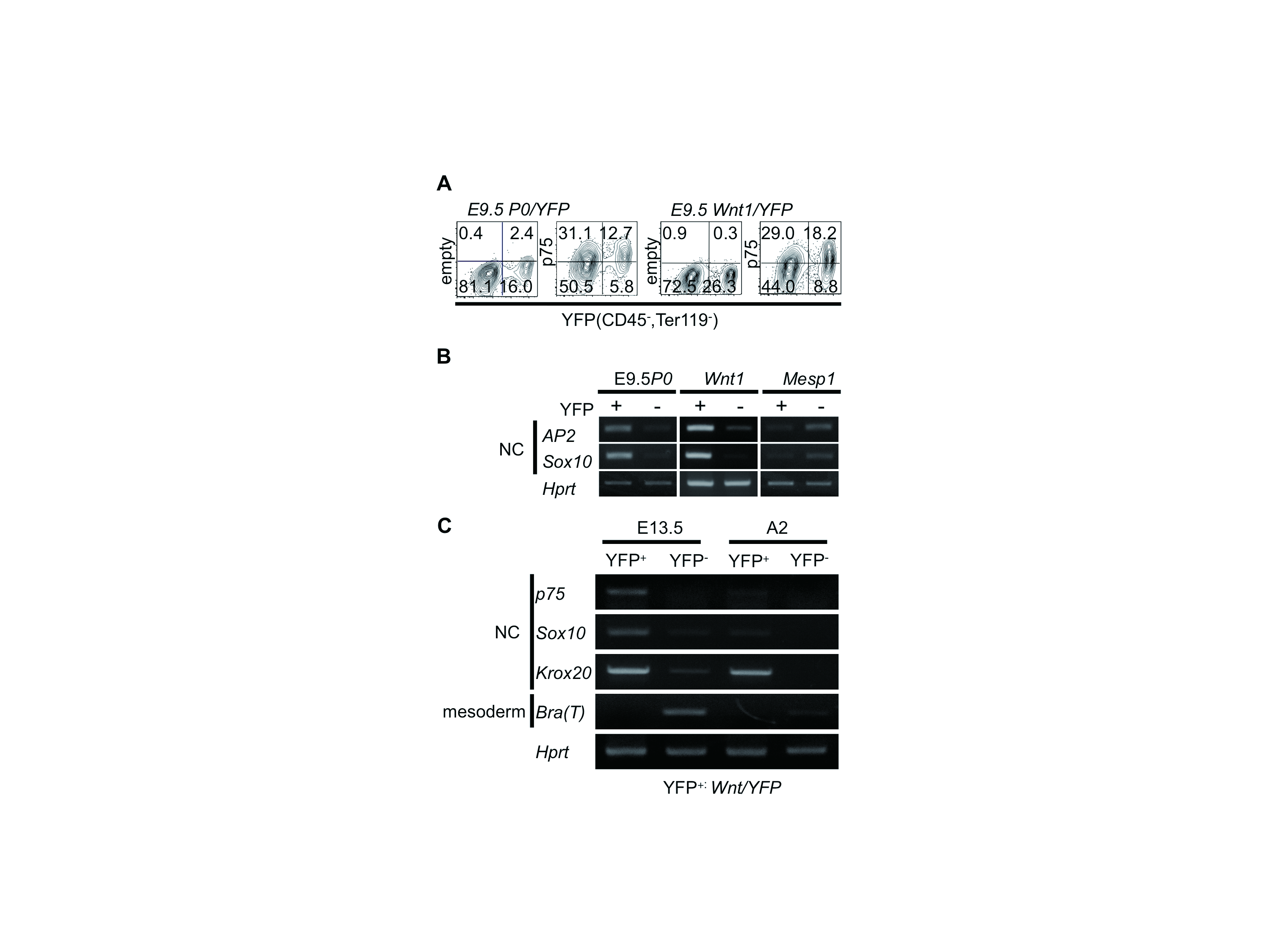

Supplement: Figure S1 — Expression of NC- and mesoderm-associated genes on cells from P0-Cre/YFP , Wnt1-Cre/YFP , and Mesp1-Cre/YFP mice. (A) Expression of p75NGFR on cells in the CD45− and Ter119− fractions from E9.5 P0-Cre/YFP and Wnt1-Cre/YFP embryos. Empty means secondary antibody only (Alexafluor 405-conjugated goat anti-rabbit IgG) without primary antibody. (B) Expression of NC-associated genes on cells from E9.5 P0-Cre/YFP, Wnt1-Cre/YFP, and Mesp1-Cre/YFP embryos (n = 4/group). (C) Expression of NC- and mesoderm-associated genes in dental mesenchymal cells from Wnt1-Cre/YFP mice (n = 4/group). YFP+ and YFP− cells were isolated using a cell sorter. RT-PCR was performed using RNA from these cells. Hypoxanthine guanidine phosphoribosyl transferase (Hprt) was the positive control; no expression was detected without a template (data not shown). (TIF) [file pone.0046436.s001.tif]

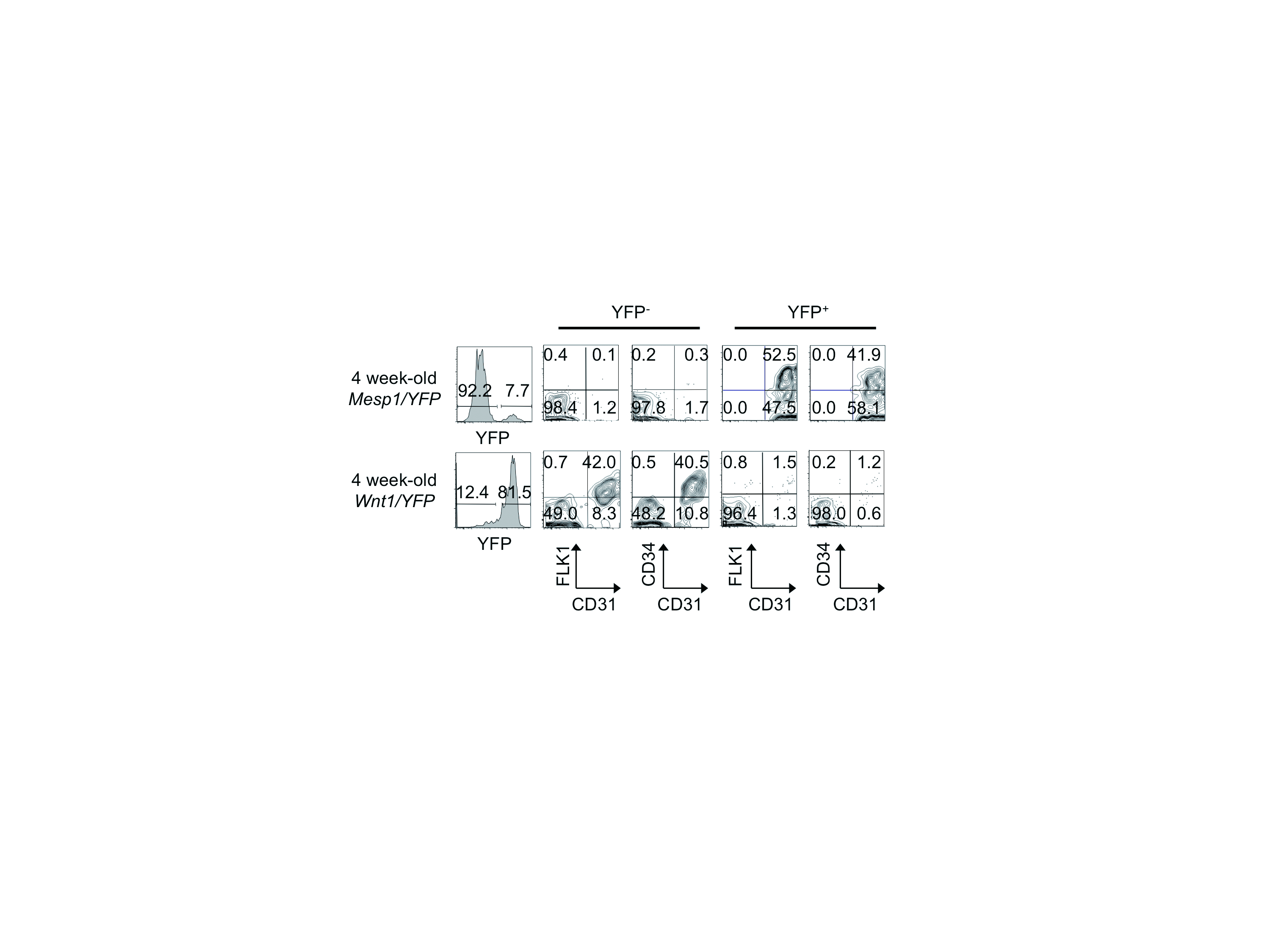

Supplement: Figure S2 — Expression of cell-surface antigens related to endothelial cells on dental mesenchymal cells from 4-week-old Wnt1-Cre/YFP and Mesp1-Cre/YFP mice. Expression of cell-surface antigens related to endothelial cells on YFP+ or YFP− dental mesenchymal cells in CD45− and Ter119− fractions from 4-week-old Wnt1-Cre/YFP and Mesp1-Cre/YFP mice. The experiments were repeated twice and one representative experiment is presented. (TIF) [file pone.0046436.s002.tif]

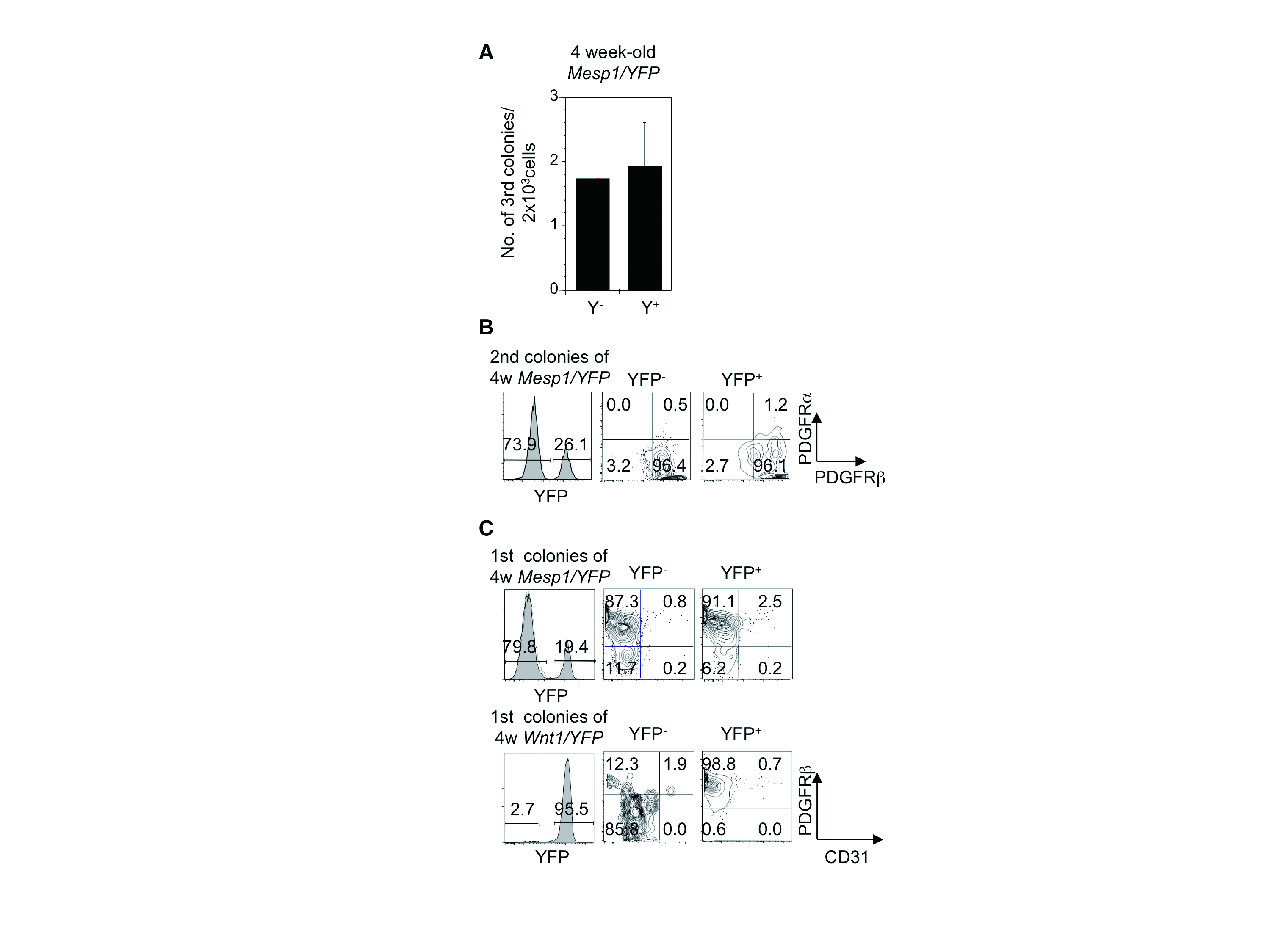

Supplement: Figure S3 — Expression of PDGFR and CD31 on dental mesenchymal cells from CFU-F progenies of 4-week-old Mesp1-Cre/YFP or Wnt1-Cre/YFP mice. (A) Number of colonies in the tertiary CFU-F assay using YFP− and YFP+ dental mesenchymal cells isolated from secondary CFU-F progenies from Mesp1-Cre/YFP mice. Values represent the mean (SD) of triplicate cultures. (B) Expression of PDGFRa and PDGFRβ on YFP− and YFP+ dental mesenchymal cells from secondary CFU-F progenies from Mesp1-Cre/YFP mice. (C) Expression of CD31 and PDGFRβ on YFP+ and YFP− cells recovered from primary CFU-F progenies from Mesp1-Cre/YFP and Wnt1-Cre/YFP mice. The experiments were repeated twice and one representative experiment is presented. (TIF) [file pone.0046436.s003.tif]

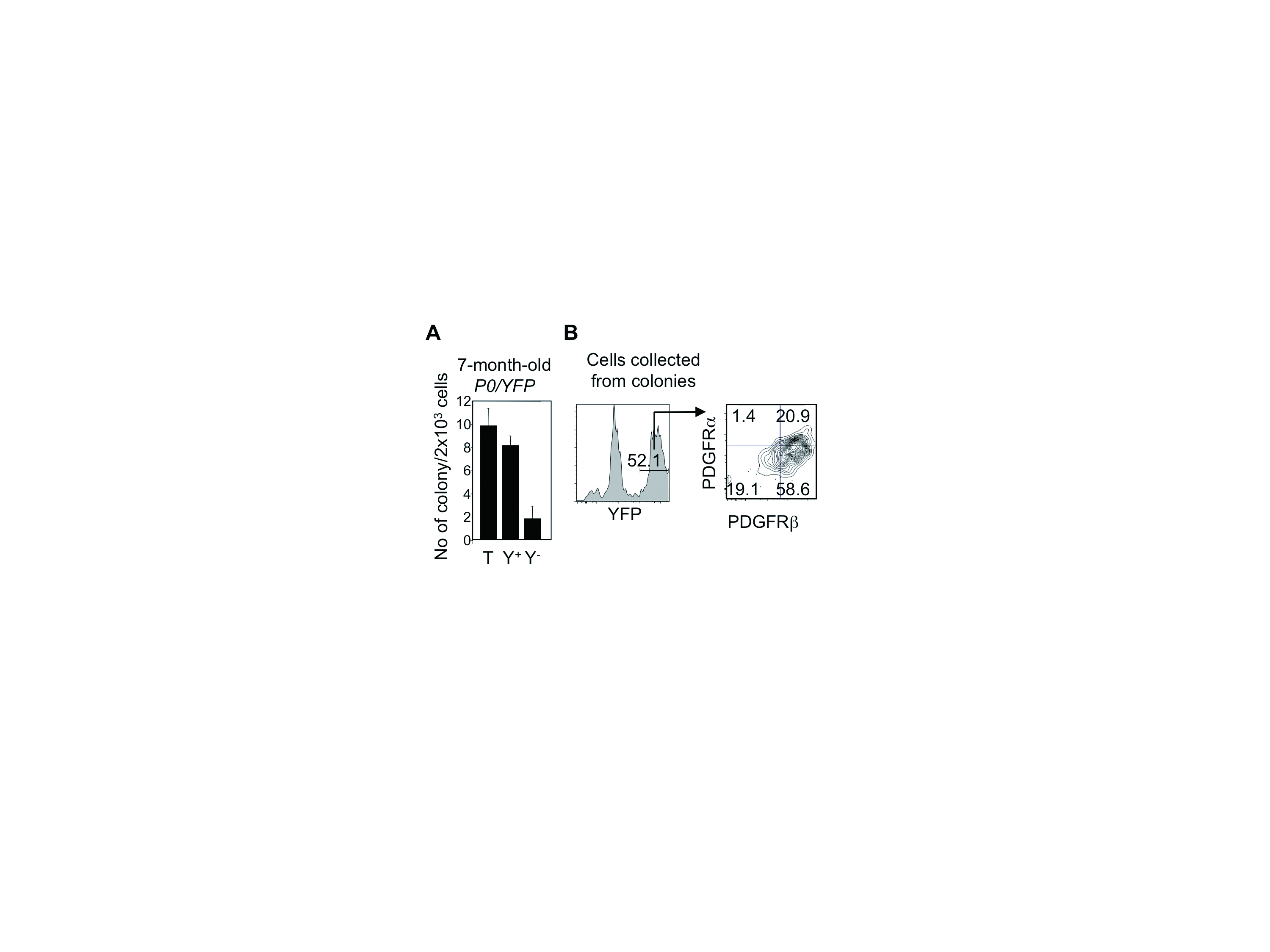

Supplement: Figure S4 — CFU-Fs of BM mesenchymal cells from 7-month-old P0-Cre/YFP mice. (A) Numbers of colonies induced from BM mesenchymal cells from 7-month-old P0-Cre/YFP mice. (B) Expression of YFP, PDGFRα, and PDGFRβ on cells from these colonies. Values represent the mean (SD) of triplicate cultures. The experiments were repeated twice and one representative experiment is presented. (TIF) [file pone.0046436.s004.tif]
