# Supplementary material for: Origins and Properties of Dental, Thymic, and Bone Marrow Mesenchymal Cells and Their Stem Cells
Source: PLoS One. 2012 Nov 21;7(11):e46436. doi: 10.1371/journal.pone.0046436 (PMC3504117; doi:10.1371/journal.pone.0046436)
Supplement: Table S1 — The number of primary and secondary colonies and the origin of colony-forming cells in dental mesenchymal cells. Numbers of primary colonies (1st CFU-F, >50 cells) induced from 4×10^3^ dental mesenchymal cells from 4-week-old Wnt1-Cre/YFP and Mesp1-Cre/YFP mice. Numbers of secondary colonies (2nd CFU-F, >50 cells) induced from 1×10^3^ primary colonies of 4-week-old mice. Values represent the means (SD) of triplicate cultures. Asterisks indicate total number of colonies obtained from triplicate cultures of two independent experiments. (DOC) [file pone.0046436.s005.doc]

| Table S1. The number of primary and secondary colonies and the origin of colony-forming cells in dental mesenchymal cells. | | | | |
| --- | --- | --- | --- | --- |
|  |  |  |  |  |
| CFU-F | No of colony | Frequency of colony (%) | No of YFP^+^ colony/ Total colony | % of YFP^+^ colony/total colony |
| Primary CFU-F |  |  |  |  |
| 4W  *Wnt1-Cre/YFP (Exp1)* | 4.0±0.9 | 0.10 | (30/30)* | 100 |
| 4W  *Mesp1-Cre/YFP (Exp1)* | 2.5±0.5 | 0.06 | (0/27)* | 0 |
| 4W  *Wnt1-Cre/YFP (Exp2)* | 5.3±1.6 | 0.13 | (42/43)* | 97.7 |
| 4W  *Mesp1-Cre/YFP (Exp2)* | 11.5±2.0 | 0.29 | (4/31) | 12.9 |
|  |  |  |  |  |
| Secondary CFU-F |  |  |  |  |
| 4W  *Wnt1-Cre/YFP (Exp1)* | 5.0±1.0 | 0.50 | (15/15) | 100 |
| 4W  *Mesp1-Cre/YFP (Exp1)* | 3.7±1.2 | 0.37 | (1/11) | 9.1 |
| 4W  *Mesp1-Cre/YFP (Exp2)* | 20.0±1.0 | 2.00 | (0/60) | 0 |
|  |  |  |  |  |
