# Supplementary material for: Origins and Properties of Dental, Thymic, and Bone Marrow Mesenchymal Cells and Their Stem Cells
Source: PLoS One. 2012 Nov 21;7(11):e46436. doi: 10.1371/journal.pone.0046436 (PMC3504117; doi:10.1371/journal.pone.0046436)
Supplement: Table S2 — Effects of inhibitory antibodies against PDGFRs in CFU-F assays using dental mesenchymal cells. Numbers of colonies were induced from 8×10^3^ dental mesenchymal cells prepared from 4-week-old Wnt1-Cre/YFP mice in the presence of inhibitory antibody against PDGFRα (APA5) and/or inhibitory antibody against PDGFRβ (APB5). No add means no antibody, and control means isotype-matched control antibody (ACK4). All antibodies were used in 10 mg/ml. Numbers of large (L, >50 cells), small (clusters) (S, <50 cells), and total colonies (T, L+S colonies) are shown. Values represent the means (SD) of triplicate cultures. Asterisks indicate a significant difference from the number of colonies in the presence of the isotype-matched control antibody (p<0.05). The experiments were repeated twice and one representative experiment is presented. (DOC) [file pone.0046436.s006.doc]

Table S2. Effects of inhibitory antibodies against PDGFRs in CFU-F assays using dental mesenchymal cells.

| Antibody | No. of colony | | | No. of Large colony | | | No. of YFP^+^ colony | | | % of YFP^+^colony/  Total colony | | % of Total (Large) colony/no add | |
| --- | --- | --- | --- | --- | --- | --- | --- | --- | --- | --- | --- | --- | --- |
| No add | 9.0 | ± | 1.0 | 8.7 | ± | 0.6 |  |  |  | NT |  | 100(100) |  |
| control | 11.3 | ± | 1.5 | 9.0 | ± | 1.0 | 11.0 | ± | 1.0 | 97.3 |  | 125.5(103.4) |  |
| anti-PDGFRα | 10.0 | ± | 2.6 | 10.0 | ± | 2.6 | 9.3 | ± | 3.1 | 93.0 |  | 111.1(111.1) |  |
| anti-PDGFRβ | 5.0* | ± | 1.0 | 1.3* | ± | 1.5 | 5.0 | ± | 1.0 | 100.0 |  | 55.5(14.9) |  |
| anti-PDGFRα /PDGFRβ | 5.0* | ± | 1.0 | 2.0* | ± | 1.0 | 4.7 | ± | 1.2 | 94.0 |  | 55.5(22.9) |  |
|  |  |  |  |  |  |  |  |  |  |  |  |  |  |
